# Social Media Use in the United States: Implications for Health Communication

**DOI:** 10.2196/jmir.1249

**Published:** 2009-11-27

**Authors:** Wen-ying Sylvia Chou, Yvonne M Hunt, Ellen Burke Beckjord, Richard P Moser, Bradford W Hesse

**Affiliations:** ^4^Behavioral Research ProgramNational Cancer InstituteBethesdaMDUSA; ^3^RAND CorporationPittsburghPAUSA; ^2^Health Communication and Informatics Research BranchNational Cancer InstituteBethesdaMDUSA; ^1^Cancer Prevention Fellowship ProgramNational Cancer InstituteBethesdaMDUSA

**Keywords:** Internet, social media, social networking, demography, population surveillance, eHealth, new technologies, health communication

## Abstract

**Background:**

Given the rapid changes in the communication landscape brought about by participative Internet use and social media, it is important to develop a better understanding of these technologies and their impact on health communication. The first step in this effort is to identify the characteristics of current social media users. Up-to-date reporting of current social media use will help monitor the growth of social media and inform health promotion/communication efforts aiming to effectively utilize social media.

**Objective:**

The purpose of the study is to identify the sociodemographic and health-related factors associated with current adult social media users in the United States.

**Methods:**

Data came from the 2007 iteration of the Health Information National Trends Study (HINTS, N = 7674). HINTS is a nationally representative cross-sectional survey on health-related communication trends and practices. Survey respondents who reported having accessed the Internet (N = 5078) were asked whether, over the past year, they had (1) participated in an online support group, (2) written in a blog, (3) visited a social networking site. Bivariate and multivariate logistic regression analyses were conducted to identify predictors of each type of social media use.

**Results:**

Approximately 69% of US adults reported having access to the Internet in 2007. Among Internet users, 5% participated in an online support group, 7% reported blogging, and 23% used a social networking site. Multivariate analysis found that younger age was the only significant predictor of blogging and social networking site participation; a statistically significant linear relationship was observed, with younger categories reporting more frequent use. Younger age, poorer subjective health, and a personal cancer experience predicted support group participation. In general, social media are penetrating the US population independent of education, race/ethnicity, or health care access.

**Conclusions:**

Recent growth of social media is not uniformly distributed across age groups; therefore, health communication programs utilizing social media must first consider the age of the targeted population to help ensure that messages reach the intended audience. While racial/ethnic and health status–related disparities exist in Internet access, among those with Internet access, these characteristics do not affect social media use. This finding suggests that the new technologies, represented by social media, may be changing the communication pattern throughout the United States.

## Introduction

From 2005 to 2009, participation in social networking sites more than quadrupled [1]. In the health communication community, there is a widespread assumption that recent advances in Internet technologies (Web 2.0), particularly the participative Internet (known as social media), have transformed the pattern of communication, including health-related communications [2]. For example, social scientists observed that social media have increased individuals’ connectivity and enabled users’ direct participation. This observation is believed to have direct implications for health communication programs, prompting efforts to identify new opportunities of using social media to impact population health [3-6]. While these observations on the impact of social media are important in public health, little of the research in this area has been based on large-scale population data, partly due to the rapidity of technological changes. The key questions that remain unanswered include the following: (1) What is the true reach and impact of social media among the current US population? (2) What are the user characteristics of the different types of social media currently being used? Although market research has previously reported on the overall prevalence of Internet and social media use, with the exception of online support group use, user characteristics of social media have not been comprehensively examined using a nationally representative population sample [7]. Developing an empirically based understanding of these behaviors and their implications has become a key priority in current health communication research.

Given that key aims of social media research are to monitor its growth and to inform health promotion efforts aiming to utilize new communication technologies, it is important to explore the relationship between social media use and health-related factors. Current research on the relationship between social media and health has produced conflicting results. On the one hand, studies have found that social media may bear health-enhancing potential through several mechanisms. First, the Internet-based social networks may increase perceived social support and interconnectivity among individuals [8,9]. Second, with the increase of user-generated content, information sharing is seen as more democratic and patient controlled, enabling users to exchange health-related information that they need and therefore making the information more patient/consumer-centered [10]. Third, in recent times, public health programs have demonstrated success in adapting social media as a communication platform for health promotion efforts such as smoking cessation and dietary interventions, increasing their reach through cyberspace [3,4,6,11-13].

Yet, indirect and sometimes unintended negative health impacts of social media have also been identified. First, the participatory nature of social media entails an open forum for information exchange, therefore increasing the possibility of wide dissemination of noncredible, and potentially erroneous, health information [14,15]. Second, health scientists exploring the issue of the digital divide have found evidence of a double divide. Specifically, those without Internet access (a large portion of whom may be without adequate health care access) are prevented from gaining health information available on the Internet [16-20]. In sum, given the direct and indirect health impacts and the wide range of and divergent results, the current study will offer an opportunity to disentangle aspects of the complex relationship between social media use and health-related factors.

The most recent iteration of the Health Information National Trends Survey (HINTS 2007) is an ideal data source to provide an in-depth examination of the prevalence and user characteristics of social media. This nationally representative survey is uniquely positioned to study social media because this new iteration contains specific follow-up questions for all Internet users, allowing us to separately estimate and compare the use of different types of social media. Another distinct advantage of the HINTS 2007 is its inclusion of many health-related questions, enabling us to comprehensively examine the association between social media use and several important health proxies. Our primary research aims are to (1) report on the prevalence of three forms of social media use in 2007: online support group participation, blogging, and social networking site participation; and (2) identify the sociodemographic and health-related predictors of the use of these three forms of social media.

## Methods

### Data Source

The data for this study were drawn from HINTS 2007, developed by the National Cancer Institute in 2007 with data collected from January 2008 through May 2008. Publicly accessible on the Internet, the HINTS is a biennial national survey of the US civilian noninstitutionalized adult population designed to assess the American public’s use of health- and cancer-related information and to assess other cancer-related knowledge, attitudes, and behaviors. The survey’s primary goal is to inform social scientists and program planners about current health communication usage across populations and to assist in developing effective health communication strategies in an age of rapid communication changes. Comprehensive reports on the conceptual framework and sample design of HINTS are published elsewhere [21,22]. Note that while the conceptual framework and most survey content remained consistent across the three iterations of HINTS (2003, 2005, and 2007), the newest iteration (HINTS 2007) contains some changes. Detailed information about HINTS 2007 scope and methodology can be found in a comprehensive report [23]. Specifically, in addition to the inclusion of new survey items (such as items concerning blogging and social networking site participation), a new sampling method was adopted for HINTS 2007 to increase response rates and reduce bias. Two modes were used for data collection: (1) a random digit dial telephone survey, using a computer-assisted telephone interview, of representative samples of US households with land-line telephones (N = 4092); and (2) a pencil-and-paper questionnaire mailed to representative US postal addresses that oversampled for minorities (N = 3582). The use of the dual sampling frames was a response to the recent dramatic decrease in telephone survey response rates and is a method currently being utilized by other government agencies. Response rates were 24% for the random digit dial survey and 31% for the mail survey. Complete surveys were obtained from 7674 adults. Only Internet users (N = 5078; approximately 68% of the population) were asked about social media use, and they form the sample for the current study.

HINTS contained both final sample weights that helped obtain population-level estimates and a set of 50 replicate sampling weights to obtain the correct standard errors; both of these were included in the present analysis. Detailed descriptions of how the sample and replicate weights were calculated can be found in the HINTS 2007 Final Report [23].

### Study Variables

We selected the following sociodemographic variables to be included in the study: age, gender, education, and race/ethnicity. Age was categorized into six groups: 18-24, 25-34, 35-44, 45-54, 55-64, 65 and above. Education was categorized as high school degree or less, some college, or college graduate. Race/ethnicity was coded into one of the following four categories: non-Hispanic white, non-Hispanic black (black/African American), Hispanic, and non-Hispanic other.

In addition to the sociodemographic variables, three health-related variables were examined. The first is self-described health status, including overall health and distress level(measured by a summed score of six-item assessment of depressive symptoms borrowed from the National Health Interview Survey, 1997, Adult Core Questionnaire [24]). The second is the respondent’s cancer experience, coded into three categories: (1) having had a personal diagnosis of cancer, (2) having had a family member diagnosed with cancer, or (3) having had no personal experience or family member with cancer. Note that the categories are mutually exclusive: individuals with a personal diagnosis of cancer are automatically categorized as (1) regardless of their status in (2). The final health-related variable is health care access, measured by whether the respondent reports having a regular health care provider or not.

Internet status was measured by response to the following question: “Do you ever go on-line to access the Internet or World Wide Web, or to send and receive an email?” Among Internet users, social media use was assessed by responses to the following three questions: “In the past 12 months, have you done the following while using the Internet: (1) participated in an on-line support group for people with a similar health or medical issue? (2) wrote in an online diary or blog? (3) visited a social networking site, such as ‘My Space’ or ‘Second Life’?”

### Data Analysis

To accommodate the complex sampling design of HINTS, analyses were conducted using SUDAAN, version 10 (Research Triangle Institute, Research Triangle Park, NC, USA). Missing data (with responses of “refuse” or “don't know”) were recoded as missing for all analyses. Bivariate analyses (chi-square) were conducted to estimate the prevalence of social media use and associations between study variables and each of the three types of social media. To address potential differences in responses due to the dual frames of the 2007 survey, we tested for potential mode differences and found no differential responses by mode to any of the social media use outcomes of interests; thus, a combined sample was used for subsequent analysis.

Separate multivariate logistic regression models were conducted to estimate the odds of writing a blog, participating in an online support group, and participating in a social networking site, while including a set of demographic and health-related predictors. Finally, given the overwhelmingly significant contribution of age in all three models, each outcome was tested using age-stratified analyses by running separate models within each of the three age categories of 18-34, 35-54, and 55 and above.

## Results

### Sample Characteristics

In 2007, approximately 69% of the US population reported having access to the Internet. This estimate is consistent with other prevalence estimates of Internet use in the same period [1]. Table 1 displays the weighted sample characteristics of non-Internet users and Internet users.

**Table 1:** Weighted sample characteristics: proportion of non-Internet and Internet users in each category

Characteristic	Non-Internet Users (N = 2566, 31.46%)	Internet Users (N = 5078, 68.54%)
**Age**	*P* < .001	
18-24	15.2%	84.8%
25-34	23.0%	77.0%
35-44	21.7%	78.3%
45-54	28.9%	71.1%
55-64	33.0%	67.0%
65+	66.4%	33.6%
**Gender**	*P* = .003	
Male	33.6%	66.4%
Female	29.5%	70.6%
**Education**	*P* < .001	
High school or less	50.5%	49.5%
Some college	17.1%	82.9%
College graduate	9.0%	91.0%
**Race/ethnicity**	*P* < .001	
Non-Hispanic white	25.0%	75.2%
Hispanic	50.7%	49.3%
Black/African American	43.3%	56.8%
Other^a^	25.8%	74.2%
**General health**	*P* < .001	
Excellent, very good, or good	26.7%	73.3%
Fair or poor	51.4%	48.6%
**Psychological distress**	*P* < .001	
Yes	43.4%	56.6%
No	28.4%	71.6%
**Cancer experience**	*P* < .001	
No personal experience with cancer	36.7%	63.3%
Had family with cancer	26.3%	73.7%
Cancer survivor	43.1%	56.9%
**Have regular health care provider**	*P* < .001	
Yes	29.1%	70.9%
No	35.7%	64.3%

^a^ Other includes American Indian, Asian American, Pacific Islander, Native Hawaiian, Alaskan Native, and multiple races mentioned.

Bivariate analyses revealed a number of significant differences between Internet users and non-Internet users. Consistent with prior results, non-Internet users were more likely to be ethnic minorities, older, less educated, less healthy, more distressed, and to report a history of a cancer diagnosis.

Further, as Table 2 below shows, among Internet users, approximately 27% reported using at least one form of social media. We used chi-square tests to compare those who reported using social media (as defined by individuals who responded “yes” to at least one of the three questions on social media) to Internet users who reported not using social media.

**Table 2:** Weighted sample characteristics of Internet users (N = 5078, 68.54% of US population) who use and do not use social media

Characteristic	Don’t Use Social Media (N = 3660, 72.65%)	Use Social Media (N = 1378, 27.35%)
**Age^a^**	*P* < .001	
18-24	23.7%	76.4%
25-34	42.7%	57.3%
35-44	64.6%	35.5%
45-54	77.6%	22.4%
55-64	86.8%	13.1%
65+	92.0%	8.00%
**Gender**	*P* = .25	
Male	60.2%	39.8%
Female	62.6%	37.4%
**Education^a^**	*P* = .02	
High school or less	62.1%	37.9%
Some college	58.7%	41.3%
College graduate	65.3%	34.7%
**Race/ethnicity^a^**	*P* < .001	
Non-Hispanic White	64.9%	35.2%
Hispanic	56.4%	43.6%
Black/African American	53.4%	46.6%
Other^b^	49.7%	50.3%
**General health**	*P* = .27	
Excellent, very good, or good	62.5%	37.5%
Fair or poor	58.3%	41.7%
**Psychological distress^a^**	*P* = .02	
Yes	49.1%	50.9%
No	62.7%	37.3%
**Cancer experience**^a^	*P* < .001	
No personal experience with cancer	61.3%	38.7%
Had family with cancer	60.7%	39.3%
Cancer survivor	81.6%	18.4%
**Have regular health care provider^a^**	*P* < .001	
Yes	65.4%	34.7%
No	52.4%	47.6%

^a^ Variables that are significantly associated with social media use at *P* < .05 level.

^b^ Other includes American Indian, Asian American, Pacific Islander, Native Hawaiian, Alaskan Native, and multiple races mentioned.

Among Internet users, use of social media was not uniformly distributed across the age strata. The largest proportion of social media use occurred among Internet users between the ages of 18 and 24 (65%) and decreased thereafter with each subsequent age group. In addition, patterns of social media use varied by race. Non-white Americans who accessed the Internet were more likely to use social media than white Americans.

The potentially different user characteristics among different types of social media prompted separate analyses by each type of media. Table 3 summarizes the bivariate associations between each type of social media (not mutually exclusive) and the study variables.

**Table 3:** Bivariate associations between three types of social media use and study variables: weighted results

Characteristic	Online Support Group Users (N = 232)	Bloggers (N = 356)	Social Networking Site Users (N = 1159)
**Percent of Internet users**	4.6%	7.1%	23.0%
**Age^a^**	*P* < .001	*P* < .001	*P* < .001
18-24	1.4%	21.3%	74.0%
25-34	7.8%	16.3%	52.1%
35-44	6.7%	8.2%	30.4%
45-54	5.3%	4.7%	17.5%
55-64	3.6%	3.2%	9.2%
65+	2.0%	1.3%	5.5%
**Gender**	*P* = .06	*P* = .34	*P* = .13
Male	4.0%	9.3%	35.9%
Female	5.9%	10.6%	32.7%
**Education^a^**	*P* = .02	*P* = .12	*P* = .005
High school or less	3.5%	8.4%	35.4%
Some college	6.7%	12.0%	36.8%
College graduate	4.2%	8.8%	29.7%
**Race/ethnicity**^a^	*P* = .81	*P* = .43	*P* < .001
Non-Hispanic white	5.0%	8.9%	31.2%
Hispanic	3.5%	9.1%	41.3%
Black/African American	5.2%	12.9%	42.8%
Other^b^	4.9%	12.9%	44.7%
**General health^a^**	*P* = .003	*P* = .82	*P* = .70
Excellent, very good, or good	4.1%	9.7%	33.8%
Fair or poor	10.8%	10.2%	35.2%
**Psychological distress^a^**	*P* = .01	*P* = .45	*P* = .06
Yes	15.7%	13.5%	44.6%
No	4.2%	9.6%	33.4%
**Cancer experience^a^**	*P* < .001	*P* < .001	*P* < .001
No personal experience with cancer	2.6%	8.1%	36.5%
Have family with cancer	5.4%	11.0%	35.0%
Cancer survivor	8.1%	3.5%	10.2%
**Have regular health care provider**^a^	*P* = .83	*P* = .02	*P* < .001
Yes	5.1%	8.6%	30.2%
No	4.7%	13.3%	43.7%

^a^ Variables that are significantly associated with one or more of the social media variables at *P* < .05 level.

^b^ Other includes American Indian, Asian American, Pacific Islander, Native Hawaiian, Alaskan Native, and multiple races mentioned.

Among the three forms of social media included in the survey, social networking received the most utilization (23% of Internet users), followed by blogging (7% of Internet users) and, finally, participation in online support groups (5% of Internet users).

Blogging and social networking site participation showed the expected inverse linear relationship with age (ie, increased use across decreasing age strata). Partially because of the younger age, users tend to not have personal experience with cancer and not have a regular health care provider. The user characteristic profile of online support group participation was distinct from the other two forms of social media. Use of online support groups was rarely seen in the youngest age group (18-24) and was uniquely associated with several health-related factors, including rating general health as poor and reporting psychological distress. In contrast, blogging and social networking site participation were not associated with measures of self-reported health status. Finally, we found an unexpected education and racial/ethnic breakdown among social networking site users: less-educated individuals and racial/ethnic minorities were more likely to use this form of social media. However, these differences disappeared in subsequent regression analyses (below), suggesting that the differences observed here are likely explained by age.

### Multivariate Analyses

The three separate multivariate regressions estimated the odds of using a particular form of social media in HINTS 2007. Given that gender was not associated with social media use at the bivariate level, we dropped it from the regression models. Table 4 displays the results of the analysis.

Among Internet users, online support group participation was predicted by age, education, as well as several health-related factors. Compared with people 65 and over, those aged 25-44 were three to five times more likely to use support groups. Compared with college graduates, those with some college were more likely to use support groups. Moreover, consistent with the bivariate-level observations, those who regarded themselves as less healthy, more distressed, and who had a personal cancer experience were more likely to have used online support groups, confirming that health status is an important determinant of support group participation.

In contrast to the model for support group participation, age emerged as the only significant predictor in the models of blogging and social networking site participation. A statistically significant linear effect of age on the two outcome variables was observed (*P* < .001). Among individuals aged 55 and below, we observed a strong linear age effect, with each decreasing age stratum, in the odds of blogging. Participation in social networking sites shared similar user characteristics, except the odds ratios were even larger, with the age effect encompassing every age stratum. In addition, among Internet users, African Americans were more likely than non-Hispanic whites to use a social networking site (OR = 1.51, 95% CI 1.01-2.24).

**Table 4:** Multivariate logistic regressions of three types of social media use among Internet users

Characteristic	Odds of Participating in an Online Support Group		Odds of Writing in a Blog		Odds of Using a Social Networking Site	
	OR (95% CI)	*P*	OR (95% CI)	*P*	OR (95% CI)	*P*
**Age**		< .001		< .001		< .001
18-24	0.98 (0.28-3.45)	.98	19.11 (7.60-48.06)	< .001	47.85 (27.92-82.00)	< .001
25-34	4.97 (2.30-10.75)	< .001	13.12 (5.53-31.13)	< .001	17.62 (10.56-29.42)	< .001
35-44	3.64 (1.87-7.08)	< .001	6.71 (2.80-16.06)	< .001	6.97 (4.57-10.64)	< .001
45-54	3.16 (1.59-6.28)	.002	3.31 (1.31-8.39)	.01	3.41 (2.23-5.20)	< .001
55-64	1.76 (0.78-3.93)	.17	1.96 (0.77-4.99)	.15	1.62 (1.04-2.52)	.03
65+	1.00		1.00		1.00	
**Education**		.01		.07		.48
High school or less	0.83 (0.48-1.43)	.49	0.73 (0.52-1.03)	.07	0.85 (0.65-1.11)	.23
Some college	1.58 (1.06-2.36)	.02	1.11 (0.77-1.60)	.56	0.96 (0.74-1.23)	.73
College graduate	1.00		1.00		1.00	
**Race/ethnicity**		.92		.47		.13
Non-Hispanic white	1.00		1.00		1.00	
Hispanic	0.75 (0.30-1.92)	.55	0.78 (0.37-1.65)	.51	0.83 (0.53-1.31)	.42
Black/African American	0.99 (0.48-2.08)	.98	1.58 (0.85-2.95)	.14	1.51 (1.01-2.24)	.04
Other^a^	1.08 (0.46-2.56)	.86	1.31 (0.63-2.75)	.46	1.36 (0.82-2.27)	.23
**General health**						
Excellent, very good, or good	1.00		1.00		1.00	
Fair or poor	2.25 (1.31-3.87)	.004	1.01 (0.53-1.93)	.97	1.09 (0.77-1.55)	.63
**Psychological distress**						
Yes	3.28 (1.59-6.77)	.001	1.45 (0.53-3.96)	.46	1.49 (0.88-2.52)	.14
No	1.00		1.00		1.00	
**Cancer experience**		.002		.15		.02
No personal experience with cancer	1.00		1.00		1.00	
Have family with cancer	2.11 (1.24-3.58)	.007	1.53 (0.99-2.38)	.06	1.12 (0.85-1.48)	.41
Cancer survivor	4.20 (1.98-8.92)	< .001	1.24 (0.56-2.74)	.60	0.63 (0.40-1.01)	.06
**Have regular health care provider**						
Yes	1.05 (0.58-1.90)	.87	0.99 (0.63-1.58)	.97	1.13 (0.85-1.52)	.39
No	1.00		1.00		1.00	

^a^ Other includes American Indian, Asian American, Pacific Islander, Native Hawaiian, Alaskan Native, and multiple races mentioned.

### Age-Stratified Multivariate Analyses

Given the central role of age in predicting social media use, and the significant interactions found between age and race/ethnicity, we conducted age-stratified logistic regressions to see whether adjusting for specific age strata would illuminate other important variables associated with social media use. Age was stratified into three categories for multivariate logistic regression models: 18-34 (younger group), 35-54 (middle-age group), 55 and older (older group). In general, the stratified models confirmed age to be the single most important predictor of social media use. Significant predictors within each type are summarized below. Note that all results reported are significant at *P* < .05.

#### Online Support Group

In the youngest group, higher education (OR = 6.33, 95% CI 2.10-19.10) and higher distress level (OR = 5.56, 95% CI 1.65-18.76) explained the outcome. Among the middle-age group, female gender (OR = 2.04, 95% CI 1.20-3.46) and higher education (OR = 2.13, 95% CI 1.21-5.12) were significant predictors. In the oldest group, poorer self-reported health (OR = 3.39, 95% CI 1.38-8.34) explained support group use.

#### Blogging

In all three age categories, the age-stratified models found no significant predictors of blogging.

#### Social Networking Sites

In the middle-age group, having no personal experience with cancer predicted social networking site participation (OR = 0.39, 95% CI 0.18-0.86). For the oldest group, male gender was the sole predictor of social networking site use (OR = 1.87, 95% CI 1.28-2.71).

## Discussion

The current study examined sociodemographic and health-related predictors of the use of three forms of social media in an effort to better understand who is accessing and being reached through these emerging communication channels. The results showed that these three forms of social media have distinctly different use patterns and user characteristics, hence different health communication implications. Among the three forms of social media considered in this study, social networking sites by far attract the most users, making them an obvious target for maximizing the reach and impact of health communication and eHealth interventions. Furthermore, with increasing prevalence of personal wireless devices, communication scientists unanimously anticipate the popularity of social networking applications to continue to grow worldwide [2,25-27]. Compared to social networking sites, a much smaller percentage of Internet users reported writing in a blog, suggesting a lower prevalence of blogging. However, reading and commenting on a blog may have been a more reliable measure of blogosphere penetration due to its lower intensity than actively keeping a blog. Moreover, the blogosphere presents a tremendous opportunity for health communication. Particularly so, because bloggers have been observed to act as important communication stakeholders—not only are they information disseminators, but they play a crucial role in directing Internet traffic through opinions and hyperlinks [28].

Online support group participation was the only survey item included in the present study that was assessed throughout the three iterations of HINTS, and the weighted prevalence estimates suggest a minor increase: in 2003 and 2005, 3.9% of Internet users had participated in online support groups compared to 4.6% in 2007. User characteristics of support groups differed from blogging and social networking site participation, suggesting that online support group participation is driven by health status. This disease-focused medium may be gradually replaced by more interactive, patient-directed social networking sites and blogs, such as CaringBridge and Patientslikeme. These forms of social media have the potential to serve the social support and empowerment functions previously identified for online support groups [29].

Apart from the patterns described above, the results of the study underscore the extent to which age determines who among US adult Internet users are engaging with social media. In this nationally representative sample, age emerged as the single strongest predictor of both social networking and blogging. In light of these findings, it seems reasonable to conclude that health communication efforts utilizing social media will have the broadest reach and impact when the target population is the younger generation. The relatively low penetration in the older population of 55 and older suggests that it is not yet an opportune time to utilize social media in communication with this age group. While this is true currently, we predict a continuing increase in utilization across all generations and groups in the next few years, and it remains a key health communication priority to continue tracking the sociodemographic trends of social media use to be sure that health communicators leverage these dissemination channels most effectively. Finally, for cancer communication efforts, this study found a high prevalence of Internet and social media use among individuals with family members who have/had cancer (see Table 1 and Table 2), suggesting the potential effectiveness of social media cancer communication efforts targeting “secondary audiences,” that is, caregivers, family, and friends of cancer patients*.*

A key finding of this study offers new and important implications for health communication in this digital age: among Internet users, social media are found to penetrate the population regardless of education, race/ethnicity, or health care access. In particular, the multivariate analyses showed that having access to a regular health care provider did not predict social media use, suggesting that its significance in the bivariate analyses was primarily due to the effect of age. Specifically, younger individuals are less likely to have a regular health care provider. Considering implications of health communication efforts, the results of this study suggest that in the future, social media promise to be a way to reach the target population regardless of socioeconomic and health-related characteristics. If we can enable broader and more equitable Internet access (eg, increasing broadband access or wireless mobile access), thus reducing the digital divide, the potential for impacting the health and health behavior of the general US population through social media is tremendous. Furthermore, the results showed social networking sites are being utilized by African Americans at a higher rate than by non-Hispanic whites. Given the continuing increase in Internet penetration, these findings suggest a potential systematic shift in the communication pattern that transcends the traditional digital divide. Future studies should continue to examine the impact of changing technologies on patterns of health disparities. On the practice side of health communication, social media outlets may represent an excellent opportunity to reach traditionally underserved members of the population.

### Limitations

The nature of self-report and the current low survey response rates present two major challenges to the generalizability of the results. First, the accuracy of self-reports of specific Internet usage may be affected by recall bias and respondents’ comprehension of survey items. In spite of this issue, this study’s prevalence estimates on Internet and social media penetration are in agreement with the published literature and are the first to be drawn from a nationally representative sample. One aspect to note is that compared to market surveys such as the Pew and Manhattan Research reports, the HINTS estimates are generally more conservative. This is in part attributable to the higher sampling precision mandated for federal surveys. Second, low response rate being a challenge facing all current survey research, HINTS 2007 attempted to boost response rates and extend coverage (especially to cell phone–only households) by adapting a dual sampling frame. As a result, the addition of the mail survey helped remedy the low response rate, to increase the generalizability of the data.

An additional limitation concerns the instrumentation and questions related to blogging and social networking site participation: since neither question asked specifically about health-related use of these technologies, we cannot precisely estimate the prevalence of health-related social media use using HINTS data. Given the growing role of social media in health, future iterations of HINTS may specifically capture health-related social media use [10]. As well, the question on blogging does not capture individuals who view and comment on blogs and thus may underestimate the degree to which the American public is engaged with this activity.

Finally, with new technologies and social media continuing to evolve rapidly, these data, despite being the most updated national survey data available, may not have been able to capture some emerging social media forms (eg, Twitter and Wikipedia) and rapid changes brought on by the increasing use of personal wireless devices [27]. In order to track the public’s use of new media, future research should track different age groups’ social media adoption while identifying new forms of social media. Given that the younger age groups are likely to continue their use of social media, we would expect to see a persistent increase across the middle-age population in the near future.

### Conclusions

With the goal to develop a better understanding of social media use in the current US population, we have reported on the prevalence and user characteristics of three types of social media using the 2007 HINTS survey. While observations and theories about communication changes brought about by new technologies abound, little is supported by empirical evidence based on nationally representative data. The findings of this study contribute to the knowledge base to inform future programs aiming to utilize social media.

As we have seen, forms of social media present different opportunities for health communication efforts. In particular, social networking sites attract the largest portion of Internet users and are likely to continue to grow, making them an obvious target for maximizing the reach and impact of health communication and eHealth interventions. In addition, recent growth of social media is not uniformly distributed across age groups. New health communication programs aiming to utilize social media must first consider the age of the targeted population. The data also prompt a rethinking of the connection between technologies and health disparities since the findings point to the fact that social media are penetrating individuals of different demographics at the same rate. Opportunities for narrowing the health disparities gap exist through effective use of social media as communication and health promotion platforms. These media will not enable targeted communication messages but may have the capacity to reach a wider audience than traditional media have been able to reach.

Finally, while surveillance research such as the present project is useful for determining the reach of social media, it is less useful for assessing the impact of participation in social media use on health. To assess the multiple levels of social media impact on health, future studies need to bring in diverse disciplines and methods, including intervention studies, longitudinal cohort studies, as well as ethnographic/qualitative observations to examine the effect of the social media–driven changing communication patterns on health.

## Abbreviations

HINTS: Health Information National Trends Survey

## References

[1] Jones S, Fox S (2009). Generations online in 2009.

[2] Eysenbach Gunther (2008). Medicine 2.0: social networking, collaboration, participation, apomediation, and openness. J Med Internet Res.

[3] Vance K, Howe W, Dellavalle RP (2009). Social internet sites as a source of public health information. Dermatol Clin.

[4] Thackeray Rosemary, Neiger Brad L, Hanson Carl L, McKenzie James F (2008). Enhancing promotional strategies within social marketing programs: use of Web 2.0 social media. Health Promot Pract.

[5] Centers for Disease Control and Prevention (CDC).

[6] Norman Cameron D, McIntosh Scott, Selby Peter, Eysenbach Gunther (2008). Web-assisted tobacco interventions: empowering change in the global fight for the public's (e)Health. J Med Internet Res.

[7] Atkinson Nancy L, Saperstein Sandra L, Pleis John (2009). Using the internet for health-related activities: findings from a national probability sample. J Med Internet Res.

[8] Wangberg Silje C, Andreassen Hege K, Prokosch Hans-Ulrich, Santana Silvina Maria Vagos, Sørensen Tove, Chronaki Catharine E (2008). Relations between Internet use, socio-economic status (SES), social support and subjective health. Health Promot Int.

[9] Idriss Shereene Z, Kvedar Joseph C, Watson Alice J (2009). The role of online support communities: benefits of expanded social networks to patients with psoriasis. Arch Dermatol.

[10] Hawn Carleen (2009). Take two aspirin and tweet me in the morning: how Twitter, Facebook, and other social media are reshaping health care. Health Aff (Millwood).

[11] Khanna Prerna Mona (2008). Icyou: how social media is the new resource for online health information. Medscape J Med.

[12] Khan Sharib A, McFarlane Delano J, Li Jianhua, Ancker Jessica S, Hutchinson Carly, Cohall Alwyn, Kukafka Rita (2007). Healthy Harlem: empowering health consumers through social networking, tailoring and web 2.0 technologies. AMIA Annu Symp Proc.

[13] Block Gladys, Sternfeld Barbara, Block Clifford H, Block Torin J, Norris Jean, Hopkins Donald, Quesenberry Charles P, Husson Gail, Clancy Heather Anne (2008). Development of Alive! (A Lifestyle Intervention Via Email), and its effect on health-related quality of life, presenteeism, and other behavioral outcomes: randomized controlled trial. J Med Internet Res.

[14] Habel Melissa A, Liddon Nicole, Stryker J E (2009). The HPV vaccine: a content analysis of online news stories. J Womens Health (Larchmt).

[15] Kortum Philip, Edwards Christine, Richards-Kortum Rebecca (2008). The impact of inaccurate Internet health information in a secondary school learning environment. J Med Internet Res.

[16] Viswanath K, Kreuter Matthew W (2007). Health disparities, communication inequalities, and eHealth. Am J Prev Med.

[17] Zhao Shanyang (2009). Teen adoption of MySpace and IM: inner-city versus suburban differences. Cyberpsychol Behav.

[18] Fogel Joshua, Ribisl Kurt M, Morgan Phyllis D, Humphreys Keith, Lyons Elizabeth J (2008). Underrepresentation of African Americans in online cancer support groups. J Natl Med Assoc.

[19] Jackson Linda A, Zhao Yong, Kolenic Anthony, Fitzgerald Hiram E, Harold Rena, Von Eye Alexander (2008). Race, gender, and information technology use: the new digital divide. Cyberpsychol Behav.

[20] Cammaerts B (2008). Critiques on the participatory potentials of Web 2.0. communication. Culture & Critique.

[21] Nelson DE, Kreps GL, Hesse BW, Croyle RT, Willis G, Arora NK (2004). The Health Information National Trends Survey (HINTS): development, design, and dissemination. J Health Commun.

[22] Hesse BW, Moser RP, Rutten LJF, Kreps GL (2006). The Health Information National Trends Survey: research from the baseline. J Health Commun.

[23] Cantor D, Coa K, Crystal-Mansour S, Davis T, Dipko S, Sigman R (2009). Health Information National Trends Survey (HINTS) 2007 Final Report.

[24] Beckjord Ellen Burke, Finney Rutten Lila J, Squiers Linda, Arora Neeraj K, Volckmann Lindsey, Moser Richard P, Hesse Bradford W (2007). Use of the internet to communicate with health care providers in the United States: estimates from the 2003 and 2005 Health Information National Trends Surveys (HINTS). J Med Internet Res.

[25] Della Lindsay J, Eroglu Dogan, Bernhardt Jay M, Edgerton Erin, Nall Janice (2008). Looking to the future of new media in health marketing: deriving propositions based on traditional theories. Health Mark Q.

[26] Bernhardt Jay M (2006). Improving health through health marketing. Prev Chronic Dis.

[27] Horrigan J (2009). Wireless Internet use.

[28] Lefebvre RC (2007). The new technology: the consumer as participant rather than target audience. Social Marketing Quarterly.

[29] van Uden-Kraan C F, Drossaert C H C, Taal E, Seydel E R, van de Laar M A F J (2009). Participation in online patient support groups endorses patients' empowerment. Patient Educ Couns.

